# (2*E*)-3-(4-Eth­oxy­phen­yl)-1-(2-methyl-4-phenyl­quinolin-3-yl)prop-2-en-1-one monohydrate

**DOI:** 10.1107/S1600536810048026

**Published:** 2010-11-24

**Authors:** S. Sarveswari, V. Vijayakumar, R. Prasath, T. Narasimhamurthy, Edward R. T. Tiekink

**Affiliations:** a Organic Chemistry Division, School of Advanced Sciences, VIT University, India; b Materials Research Centre, Indian Institute of Science, Bengaluru-560012, India; c Department of Chemistry, University of Malaya, 50603 Kuala Lumpur, Malaysia

## Abstract

The title hydrate, C_27_H_23_NO_2_·H_2_O, features an almost planar quinoline residue (r.m.s. deviation = 0.015 Å) with the benzene [dihedral angle = 63.80 (7) °] and chalcone [C—C—C—O torsion angle = −103.38 (18)°] substituents twisted significantly out of its plane. The configuration about the C=C bond [1.340 (2) Å] is *E*. In the crystal, mol­ecules related by the 2_1_ symmetry operation are linked along the *b* axis *via* water mol­ecules that form O—H⋯O_c_ and O—H⋯N_q_ hydrogen bonds (c = carbonyl and q = quinoline). A C—H⋯O inter­action also occurs.

## Related literature

For background to chalcones, see: Schröder *et al.* (1988 ▶); Schröder & Schröder (1990 ▶). For a related structure, see: Prasath *et al.* (2010 ▶).
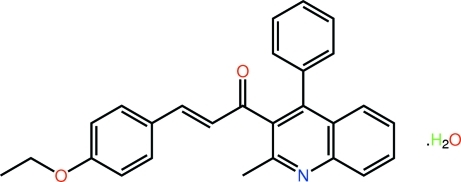


## Experimental

### 

#### Crystal data

C_27_H_23_NO_2_·H_2_O
*M* *_r_* = 411.48
Monoclinic, 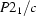

*a* = 17.4256 (4) Å
*b* = 7.6240 (2) Å
*c* = 18.4117 (4) Å
β = 116.957 (1)°
*V* = 2180.27 (9) Å^3^
*Z* = 4
Mo *K*α radiation
μ = 0.08 mm^−1^
*T* = 293 K
0.37 × 0.24 × 0.15 mm

#### Data collection

Bruker SMART APEX CCD diffractometer
Absorption correction: multi-scan (*SADABS*; Bruker, 1998 ▶) *T*_min_ = 0.977, *T*_max_ = 0.988
29183 measured reflections
4993 independent reflections
3568 reflections with *I* > 2σ(*I*)
*R*_int_ = 0.033

#### Refinement

*R*[*F* ^2^ > 2σ(*F* ^2^)] = 0.050
*wR*(*F* ^2^) = 0.146
*S* = 1.05
4993 reflections
288 parameters
3 restraints
H atoms treated by a mixture of independent and constrained refinement
Δρ_max_ = 0.25 e Å^−3^
Δρ_min_ = −0.19 e Å^−3^

### 

Data collection: *SMART* (Bruker, 2001 ▶); cell refinement: *SAINT* (Bruker, 2001 ▶); data reduction: *SAINT*; program(s) used to solve structure: *SHELXS97* (Sheldrick, 2008 ▶); program(s) used to refine structure: *SHELXL97* (Sheldrick, 2008 ▶); molecular graphics: *ORTEP-3* (Farrugia, 1997 ▶) and *DIAMOND* (Brandenburg, 2006 ▶); software used to prepare material for publication: *publCIF* (Westrip, 2010 ▶).

## Supplementary Material

Crystal structure: contains datablocks global, I. DOI: 10.1107/S1600536810048026/hb5746sup1.cif

Structure factors: contains datablocks I. DOI: 10.1107/S1600536810048026/hb5746Isup2.hkl

Additional supplementary materials:  crystallographic information; 3D view; checkCIF report

## Figures and Tables

**Table 1:** Hydrogen-bond geometry (Å, °)

*D*—H⋯*A*	*D*—H	H⋯*A*	*D*⋯*A*	*D*—H⋯*A*
O1w—H1w⋯N1^i^	0.83 (2)	2.11 (2)	2.934 (2)	174 (2)
O1w—H2w⋯O1	0.83 (2)	2.28 (2)	3.082 (2)	164 (3)
C26—H26b⋯O1^ii^	0.97	2.55	3.507 (3)	167

Symmetry codes: (i) 
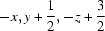
; (ii) 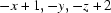.

**Fig. 1.**
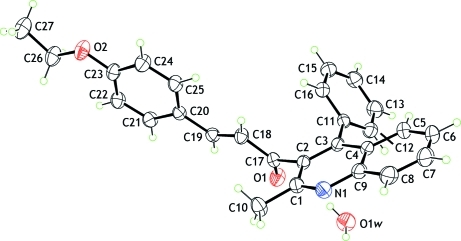
The molecular structure of (I) showing displacement ellipsoids at the 35% probability level.

**Fig. 2.**
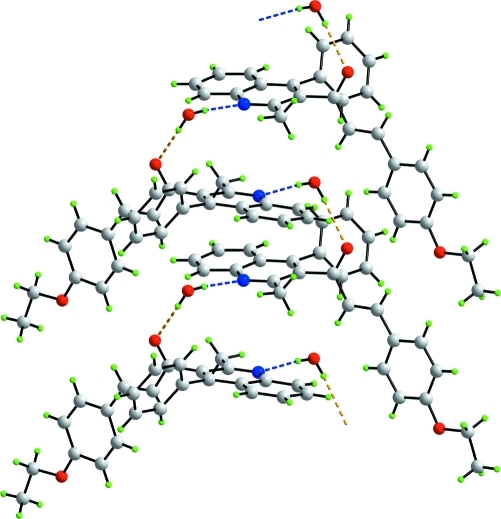
A view of a supramolecular chain sustained by O—H···O and O—H···N hydrogen bonds shown as orange and blue dashed lines, respectively.

## supplementary crystallographic information

### Comment

Chalcones are open-chain flavonoids in which two aromatic rings, jointed by a three carbon linker, are synthesized by chalcone synthetase from 3-malonyl-CoA and a starter CoA ester such as 4-coumaronyl-CoA in plants (Schröder *et al.*, 1988). Chalcone synthetase functions as a key enzyme of flavonoid biosynthesis, using the similar substrates as stilbene synthetase (Schröder & Schröder, 1990). The title hydrate, (I), was investigated in continuation of structural studies of chalcones (Prasath *et al.*, 2010).

With reference to least-squares plane through the quinoline residue in (I), Fig. 1, the phenyl ring is twisted and forms a dihedral angle of 63.80 (7) ° with it. Similarly, the chalcone residue is also twisted out of the plane as seen in the value of the C1—C2—C17—O1 torsion angle of -103.38 (18) °. There are discernible twists in the chalcone residue as seen in the value of the O1—C17—C18—C19 torsion angle of 6.8 (3) ° and especially C18—C19—C20—C21 of -168.50 (16) °. The configuration about the C18═ C19 bond [1.340 (2) Å] is *E*. The ethoxy group is lies in the plane of the benzene ring to which it is connected [C26—O2—C23—C22 = -3.7 (3) ° and C23—O2—C26—C27 = -178.30 (17) °].

The crystal packing is dominated by hydrogen bonds formed by the water molecule of crystallization. Thus, the carbonyl-O1 of one molecule is linked to a quinoline-N of another *via* O—H···O and O—H···N hydrogen bonds, Table 1. This results in the formation of a supramolecular chain with a helical topology along the *b* axis, Fig. 2. Chains are consolidated in the crystal packing by C—H···O contacts, Table 1.

### Experimental

A mixture of 3-acetyl-2-methyl-4-phenylquinoline (2.6 g 0.01 *M*) and 4-ethoxybenzaldehyde (1.5 g 0.01 *M*) and a catalytic amount of KOH in distilled ethanol (50 ml) was stirred for about 24 h. The resulting mixture was concentrated to remove ethanol then poured onto ice and neutralized with dilute acetic acid. The resultant solid was filtered off, dried and purified by column chromatography using a 1:1 mixture of ethyl acetate and petroleum ether. Recrystallization was from acetone to yield colourless blocks; Yield: 64% and m. pt: 427–429 K.

### Refinement

The C-bound H atoms were geometrically placed (C–H = 0.93–0.97 Å) and refined as riding with *U_iso_*(H) = 1.2–1.5*U_eq_*(C). The remaining H were located from a difference map and refined with O–H = 0.82±0.01 Å (with H1*w*···H2*w* = 1.36±0.015 Å), and with *U_iso_*(H) = 1.5*U_eq_*(O).

### Crystal data

**Table:**

C_27_H_23_NO_2_·H_2_O	*F*(000) = 872
*M**_r_* = 411.48	*D*_x_ = 1.254 Mg m^−^^3^
Monoclinic, *P*2_1_/*c*	Mo *K*α radiation, λ = 0.71073 Å
Hall symbol: -P 2ybc	Cell parameters from 9513 reflections
*a* = 17.4256 (4) Å	θ = 2.2–29.5°
*b* = 7.6240 (2) Å	µ = 0.08 mm^−^^1^
*c* = 18.4117 (4) Å	*T* = 293 K
β = 116.957 (1)°	Block, colourless
*V* = 2180.27 (9) Å^3^	0.37 × 0.24 × 0.15 mm
*Z* = 4	

### Data collection

**Table:**

Bruker SMART APEX CCD diffractometer	4993 independent reflections
Radiation source: fine-focus sealed tube	3568 reflections with *I* > 2σ(*I*)
graphite	*R*_int_ = 0.033
ω scans	θ_max_ = 27.5°, θ_min_ = 2.2°
Absorption correction: multi-scan (*SADABS*; Bruker, 1998)	*h* = −22→22
*T*_min_ = 0.977, *T*_max_ = 0.988	*k* = −9→8
29183 measured reflections	*l* = −18→23

### Refinement

**Table:**

Refinement on *F*^2^	Primary atom site location: structure-invariant direct methods
Least-squares matrix: full	Secondary atom site location: difference Fourier map
*R*[*F*^2^ > 2σ(*F*^2^)] = 0.050	Hydrogen site location: inferred from neighbouring sites
*wR*(*F*^2^) = 0.146	H atoms treated by a mixture of independent and constrained refinement
*S* = 1.05	*w* = 1/[σ^2^(*F*_o_^2^) + (0.065*P*)^2^ + 0.6319*P*] where *P* = (*F*_o_^2^ + 2*F*_c_^2^)/3
4993 reflections	(Δ/σ)_max_ = 0.001
288 parameters	Δρ_max_ = 0.25 e Å^−^^3^
3 restraints	Δρ_min_ = −0.19 e Å^−^^3^

### Special details

**Table:**

Geometry. All s.u.'s (except the s.u. in the dihedral angle between two l.s. planes) are estimated using the full covariance matrix. The cell s.u.'s are taken into account individually in the estimation of s.u.'s in distances, angles and torsion angles; correlations between s.u.'s in cell parameters are only used when they are defined by crystal symmetry. An approximate (isotropic) treatment of cell s.u.'s is used for estimating s.u.'s involving l.s. planes.
Refinement. Refinement of *F*^2^ against ALL reflections. The weighted *R*-factor *wR* and goodness of fit *S* are based on *F*^2^, conventional *R*-factors *R* are based on *F*, with *F* set to zero for negative *F*^2^. The threshold expression of *F*^2^ > 2σ(*F*^2^) is used only for calculating *R*-factors(gt) *etc*. and is not relevant to the choice of reflections for refinement. *R*-factors based on *F*^2^ are statistically about twice as large as those based on *F*, and *R*- factors based on ALL data will be even larger.

### Fractional atomic coordinates and isotropic or equivalent isotropic displacement parameters (Å^2^)

**Table:**

	*x*	*y*	*z*	*U*_iso_*/*U*_eq_	
O1	0.25716 (8)	0.36751 (17)	0.84346 (7)	0.0543 (3)	
O2	0.50345 (8)	−0.52976 (18)	1.12587 (8)	0.0590 (4)	
N1	−0.02026 (8)	0.1867 (2)	0.72578 (8)	0.0466 (4)	
C1	0.06079 (10)	0.1951 (2)	0.78155 (10)	0.0414 (4)	
C2	0.12999 (10)	0.2106 (2)	0.76061 (9)	0.0366 (3)	
C3	0.11343 (10)	0.2195 (2)	0.67992 (9)	0.0362 (3)	
C4	0.02593 (10)	0.2109 (2)	0.61876 (9)	0.0392 (4)	
C5	0.00012 (12)	0.2155 (2)	0.53384 (10)	0.0500 (4)	
H5	0.0416	0.2274	0.5155	0.060*	
C6	−0.08461 (13)	0.2026 (3)	0.47860 (11)	0.0593 (5)	
H6	−0.1004	0.2067	0.4231	0.071*	
C7	−0.14798 (13)	0.1831 (3)	0.50494 (12)	0.0631 (6)	
H7	−0.2055	0.1736	0.4668	0.076*	
C8	−0.12593 (12)	0.1781 (3)	0.58612 (12)	0.0574 (5)	
H8	−0.1685	0.1649	0.6030	0.069*	
C9	−0.03862 (10)	0.1928 (2)	0.64478 (10)	0.0431 (4)	
C10	0.07655 (12)	0.1904 (3)	0.86890 (11)	0.0568 (5)	
H10A	0.0225	0.1970	0.8709	0.085*	
H10B	0.1120	0.2881	0.8978	0.085*	
H10C	0.1052	0.0830	0.8937	0.085*	
C11	0.18529 (10)	0.2312 (2)	0.65688 (9)	0.0377 (4)	
C12	0.19313 (12)	0.3754 (3)	0.61469 (10)	0.0498 (4)	
H12	0.1537	0.4668	0.6010	0.060*	
C13	0.25941 (13)	0.3837 (3)	0.59282 (12)	0.0581 (5)	
H13	0.2642	0.4809	0.5646	0.070*	
C14	0.31808 (12)	0.2493 (3)	0.61263 (11)	0.0546 (5)	
H14	0.3622	0.2550	0.5975	0.066*	
C15	0.31119 (12)	0.1063 (3)	0.65488 (11)	0.0526 (5)	
H15	0.3512	0.0160	0.6688	0.063*	
C16	0.24500 (11)	0.0957 (2)	0.67697 (10)	0.0449 (4)	
H16	0.2406	−0.0018	0.7052	0.054*	
C17	0.22121 (10)	0.2250 (2)	0.82790 (9)	0.0378 (4)	
C18	0.26143 (10)	0.0642 (2)	0.87161 (9)	0.0434 (4)	
H18	0.2325	−0.0415	0.8527	0.052*	
C19	0.33811 (10)	0.0620 (2)	0.93782 (9)	0.0401 (4)	
H19	0.3659	0.1694	0.9546	0.048*	
C20	0.38267 (9)	−0.0908 (2)	0.98645 (9)	0.0375 (4)	
C21	0.45460 (10)	−0.0704 (2)	1.06181 (10)	0.0460 (4)	
H21	0.4748	0.0422	1.0799	0.055*	
C22	0.49672 (11)	−0.2115 (2)	1.11034 (10)	0.0481 (4)	
H22	0.5440	−0.1938	1.1607	0.058*	
C23	0.46812 (10)	−0.3798 (2)	1.08362 (10)	0.0449 (4)	
C24	0.39746 (11)	−0.4039 (3)	1.00726 (11)	0.0519 (4)	
H24	0.3786	−0.5167	0.9885	0.062*	
C25	0.35586 (10)	−0.2621 (2)	0.95986 (10)	0.0452 (4)	
H25	0.3090	−0.2801	0.9092	0.054*	
C26	0.57919 (12)	−0.5162 (3)	1.20221 (12)	0.0619 (5)	
H26A	0.5673	−0.4477	1.2403	0.074*	
H26B	0.6249	−0.4589	1.1948	0.074*	
C27	0.60576 (15)	−0.6989 (3)	1.23413 (14)	0.0752 (7)	
H27A	0.5606	−0.7532	1.2424	0.113*	
H27B	0.6573	−0.6941	1.2850	0.113*	
H27C	0.6163	−0.7660	1.1955	0.113*	
O1W	0.17008 (10)	0.6955 (2)	0.74149 (10)	0.0673 (4)	
H1W	0.1271 (11)	0.701 (3)	0.7493 (17)	0.101*	
H2W	0.1963 (15)	0.603 (2)	0.7613 (16)	0.101*	

### Atomic displacement parameters (Å^2^)

**Table:**

	*U*^11^	*U*^22^	*U*^33^	*U*^12^	*U*^13^	*U*^23^
O1	0.0495 (7)	0.0459 (8)	0.0520 (7)	−0.0053 (6)	0.0095 (6)	0.0020 (6)
O2	0.0491 (7)	0.0527 (8)	0.0566 (8)	0.0010 (6)	0.0077 (6)	0.0129 (6)
N1	0.0380 (7)	0.0570 (10)	0.0438 (8)	0.0002 (7)	0.0177 (6)	−0.0018 (6)
C1	0.0407 (9)	0.0450 (10)	0.0373 (8)	0.0005 (7)	0.0166 (7)	0.0011 (7)
C2	0.0360 (8)	0.0368 (9)	0.0333 (7)	0.0022 (7)	0.0123 (6)	0.0023 (6)
C3	0.0364 (8)	0.0338 (8)	0.0340 (7)	0.0034 (7)	0.0120 (6)	0.0023 (6)
C4	0.0394 (8)	0.0370 (9)	0.0350 (8)	0.0070 (7)	0.0114 (6)	0.0013 (6)
C5	0.0513 (10)	0.0555 (11)	0.0359 (8)	0.0077 (9)	0.0133 (7)	0.0014 (8)
C6	0.0595 (12)	0.0641 (13)	0.0357 (9)	0.0115 (10)	0.0053 (8)	−0.0015 (8)
C7	0.0447 (10)	0.0689 (14)	0.0505 (11)	0.0075 (10)	−0.0003 (8)	−0.0081 (10)
C8	0.0376 (9)	0.0669 (13)	0.0578 (11)	0.0040 (9)	0.0131 (8)	−0.0063 (9)
C9	0.0373 (8)	0.0435 (10)	0.0411 (8)	0.0036 (7)	0.0113 (7)	−0.0020 (7)
C10	0.0536 (11)	0.0788 (14)	0.0414 (9)	−0.0015 (10)	0.0247 (8)	0.0011 (9)
C11	0.0375 (8)	0.0431 (9)	0.0300 (7)	0.0014 (7)	0.0131 (6)	−0.0005 (6)
C12	0.0551 (10)	0.0485 (11)	0.0487 (9)	0.0076 (9)	0.0260 (8)	0.0079 (8)
C13	0.0704 (13)	0.0584 (12)	0.0568 (11)	−0.0030 (10)	0.0385 (10)	0.0072 (9)
C14	0.0504 (10)	0.0673 (13)	0.0540 (10)	−0.0019 (9)	0.0305 (9)	−0.0012 (9)
C15	0.0483 (10)	0.0583 (12)	0.0533 (10)	0.0091 (9)	0.0249 (8)	0.0023 (9)
C16	0.0473 (9)	0.0460 (10)	0.0414 (8)	0.0039 (8)	0.0201 (7)	0.0030 (7)
C17	0.0365 (8)	0.0434 (10)	0.0315 (7)	−0.0022 (7)	0.0137 (6)	0.0000 (6)
C18	0.0396 (9)	0.0433 (10)	0.0401 (8)	−0.0018 (7)	0.0117 (7)	0.0023 (7)
C19	0.0386 (8)	0.0429 (9)	0.0374 (8)	−0.0005 (7)	0.0162 (7)	−0.0025 (7)
C20	0.0315 (7)	0.0447 (9)	0.0347 (7)	0.0003 (7)	0.0135 (6)	−0.0008 (6)
C21	0.0390 (9)	0.0470 (10)	0.0422 (9)	−0.0037 (8)	0.0097 (7)	−0.0062 (7)
C22	0.0356 (8)	0.0574 (12)	0.0385 (8)	−0.0015 (8)	0.0056 (7)	−0.0011 (8)
C23	0.0363 (8)	0.0500 (11)	0.0446 (9)	0.0040 (8)	0.0151 (7)	0.0097 (8)
C24	0.0460 (10)	0.0439 (10)	0.0512 (10)	−0.0048 (8)	0.0093 (8)	0.0008 (8)
C25	0.0350 (8)	0.0509 (11)	0.0384 (8)	−0.0032 (7)	0.0067 (7)	−0.0016 (7)
C26	0.0471 (10)	0.0639 (13)	0.0558 (11)	0.0048 (10)	0.0067 (8)	0.0130 (10)
C27	0.0647 (13)	0.0706 (16)	0.0712 (14)	0.0141 (12)	0.0139 (11)	0.0229 (12)
O1W	0.0615 (9)	0.0736 (11)	0.0734 (10)	−0.0010 (8)	0.0363 (8)	0.0040 (8)

### Geometric parameters (Å, °)

**Table:**

O1—C17	1.222 (2)	C13—H13	0.9300
O2—C23	1.363 (2)	C14—C15	1.375 (3)
O2—C26	1.430 (2)	C14—H14	0.9300
N1—C1	1.318 (2)	C15—C16	1.388 (2)
N1—C9	1.377 (2)	C15—H15	0.9300
C1—C2	1.428 (2)	C16—H16	0.9300
C1—C10	1.504 (2)	C17—C18	1.460 (2)
C2—C3	1.381 (2)	C18—C19	1.340 (2)
C2—C17	1.513 (2)	C18—H18	0.9300
C3—C4	1.428 (2)	C19—C20	1.460 (2)
C3—C11	1.496 (2)	C19—H19	0.9300
C4—C9	1.415 (2)	C20—C21	1.394 (2)
C4—C5	1.418 (2)	C20—C25	1.398 (2)
C5—C6	1.365 (3)	C21—C22	1.379 (2)
C5—H5	0.9300	C21—H21	0.9300
C6—C7	1.401 (3)	C22—C23	1.383 (3)
C6—H6	0.9300	C22—H22	0.9300
C7—C8	1.365 (3)	C23—C24	1.399 (2)
C7—H7	0.9300	C24—C25	1.372 (2)
C8—C9	1.416 (2)	C24—H24	0.9300
C8—H8	0.9300	C25—H25	0.9300
C10—H10A	0.9600	C26—C27	1.501 (3)
C10—H10B	0.9600	C26—H26A	0.9700
C10—H10C	0.9600	C26—H26B	0.9700
C11—C12	1.388 (2)	C27—H27A	0.9600
C11—C16	1.392 (2)	C27—H27B	0.9600
C12—C13	1.386 (3)	C27—H27C	0.9600
C12—H12	0.9300	O1W—H1W	0.83 (2)
C13—C14	1.375 (3)	O1W—H2W	0.83 (2)
			
C23—O2—C26	118.49 (15)	C14—C15—C16	120.59 (17)
C1—N1—C9	118.87 (14)	C14—C15—H15	119.7
N1—C1—C2	122.10 (14)	C16—C15—H15	119.7
N1—C1—C10	116.33 (15)	C15—C16—C11	119.96 (16)
C2—C1—C10	121.56 (14)	C15—C16—H16	120.0
C3—C2—C1	120.30 (14)	C11—C16—H16	120.0
C3—C2—C17	120.48 (14)	O1—C17—C18	123.42 (14)
C1—C2—C17	119.18 (13)	O1—C17—C2	119.56 (14)
C2—C3—C4	118.28 (14)	C18—C17—C2	117.02 (14)
C2—C3—C11	120.97 (13)	C19—C18—C17	122.91 (16)
C4—C3—C11	120.72 (13)	C19—C18—H18	118.5
C9—C4—C5	118.18 (15)	C17—C18—H18	118.5
C9—C4—C3	117.77 (14)	C18—C19—C20	126.98 (16)
C5—C4—C3	124.04 (15)	C18—C19—H19	116.5
C6—C5—C4	121.01 (18)	C20—C19—H19	116.5
C6—C5—H5	119.5	C21—C20—C25	117.36 (15)
C4—C5—H5	119.5	C21—C20—C19	120.63 (15)
C5—C6—C7	120.40 (17)	C25—C20—C19	122.01 (14)
C5—C6—H6	119.8	C22—C21—C20	122.15 (16)
C7—C6—H6	119.8	C22—C21—H21	118.9
C8—C7—C6	120.50 (17)	C20—C21—H21	118.9
C8—C7—H7	119.8	C21—C22—C23	119.56 (15)
C6—C7—H7	119.8	C21—C22—H22	120.2
C7—C8—C9	120.31 (18)	C23—C22—H22	120.2
C7—C8—H8	119.8	O2—C23—C22	125.33 (15)
C9—C8—H8	119.8	O2—C23—C24	115.31 (16)
N1—C9—C4	122.67 (14)	C22—C23—C24	119.35 (15)
N1—C9—C8	117.72 (16)	C25—C24—C23	120.42 (17)
C4—C9—C8	119.60 (16)	C25—C24—H24	119.8
C1—C10—H10A	109.5	C23—C24—H24	119.8
C1—C10—H10B	109.5	C24—C25—C20	121.12 (15)
H10A—C10—H10B	109.5	C24—C25—H25	119.4
C1—C10—H10C	109.5	C20—C25—H25	119.4
H10A—C10—H10C	109.5	O2—C26—C27	107.53 (18)
H10B—C10—H10C	109.5	O2—C26—H26A	110.2
C12—C11—C16	119.01 (15)	C27—C26—H26A	110.2
C12—C11—C3	120.88 (15)	O2—C26—H26B	110.2
C16—C11—C3	120.11 (14)	C27—C26—H26B	110.2
C13—C12—C11	120.36 (17)	H26A—C26—H26B	108.5
C13—C12—H12	119.8	C26—C27—H27A	109.5
C11—C12—H12	119.8	C26—C27—H27B	109.5
C14—C13—C12	120.38 (18)	H27A—C27—H27B	109.5
C14—C13—H13	119.8	C26—C27—H27C	109.5
C12—C13—H13	119.8	H27A—C27—H27C	109.5
C13—C14—C15	119.71 (17)	H27B—C27—H27C	109.5
C13—C14—H14	120.1	H1W—O1W—H2W	109.0 (18)
C15—C14—H14	120.1		
			
C9—N1—C1—C2	0.1 (3)	C16—C11—C12—C13	0.1 (3)
C9—N1—C1—C10	179.15 (16)	C3—C11—C12—C13	−179.23 (16)
N1—C1—C2—C3	0.8 (3)	C11—C12—C13—C14	0.0 (3)
C10—C1—C2—C3	−178.20 (17)	C12—C13—C14—C15	−0.5 (3)
N1—C1—C2—C17	178.19 (15)	C13—C14—C15—C16	0.7 (3)
C10—C1—C2—C17	−0.8 (2)	C14—C15—C16—C11	−0.5 (3)
C1—C2—C3—C4	−0.7 (2)	C12—C11—C16—C15	0.1 (2)
C17—C2—C3—C4	−178.00 (14)	C3—C11—C16—C15	179.46 (15)
C1—C2—C3—C11	−178.44 (15)	C3—C2—C17—O1	74.0 (2)
C17—C2—C3—C11	4.2 (2)	C1—C2—C17—O1	−103.38 (18)
C2—C3—C4—C9	−0.3 (2)	C3—C2—C17—C18	−106.50 (18)
C11—C3—C4—C9	177.47 (15)	C1—C2—C17—C18	76.12 (19)
C2—C3—C4—C5	−178.84 (16)	O1—C17—C18—C19	6.8 (3)
C11—C3—C4—C5	−1.1 (2)	C2—C17—C18—C19	−172.70 (15)
C9—C4—C5—C6	−0.1 (3)	C17—C18—C19—C20	178.70 (15)
C3—C4—C5—C6	178.44 (17)	C18—C19—C20—C21	−168.50 (16)
C4—C5—C6—C7	−0.5 (3)	C18—C19—C20—C25	11.8 (3)
C5—C6—C7—C8	0.5 (3)	C25—C20—C21—C22	−2.2 (3)
C6—C7—C8—C9	0.1 (3)	C19—C20—C21—C22	178.11 (16)
C1—N1—C9—C4	−1.1 (3)	C20—C21—C22—C23	1.0 (3)
C1—N1—C9—C8	178.07 (17)	C26—O2—C23—C22	−3.7 (3)
C5—C4—C9—N1	179.87 (16)	C26—O2—C23—C24	176.17 (16)
C3—C4—C9—N1	1.3 (2)	C21—C22—C23—O2	−179.40 (17)
C5—C4—C9—C8	0.7 (2)	C21—C22—C23—C24	0.8 (3)
C3—C4—C9—C8	−177.95 (16)	O2—C23—C24—C25	178.97 (16)
C7—C8—C9—N1	−179.94 (19)	C22—C23—C24—C25	−1.2 (3)
C7—C8—C9—C4	−0.7 (3)	C23—C24—C25—C20	−0.1 (3)
C2—C3—C11—C12	−118.53 (18)	C21—C20—C25—C24	1.7 (3)
C4—C3—C11—C12	63.7 (2)	C19—C20—C25—C24	−178.55 (16)
C2—C3—C11—C16	62.1 (2)	C23—O2—C26—C27	−178.30 (17)
C4—C3—C11—C16	−115.62 (17)		

### Hydrogen-bond geometry (Å, °)

**Table:**

*D*—H···*A*	*D*—H	H···*A*	*D*···*A*	*D*—H···*A*
O1w—H1w···N1^i^	0.83 (2)	2.11 (2)	2.934 (2)	174 (2)
O1w—H2w···O1	0.83 (2)	2.28 (2)	3.082 (2)	164 (3)
C26—H26b···O1^ii^	0.97	2.55	3.507 (3)	167

Symmetry codes: (i) −*x*, *y*+1/2, −*z*+3/2;  (ii) −*x*+1, −*y*, −*z*+2.
